# MicroRNAs as biomarkers for CNS disease

**DOI:** 10.3389/fnmol.2013.00039

**Published:** 2013-11-26

**Authors:** Pooja Rao, Eva Benito, André Fischer

**Affiliations:** ^1^Department of Psychiatry and Psychotherapy, University Medical Center GöttingenGöttingen, Germany; ^2^German Center for Neurodegenerative Diseases (DZNE)Göttingen, Germany

**Keywords:** biomarker, microRNA, next-generation sequencing, CSF, plasma, exosome

## Abstract

For many neurological diseases, the efficacy and outcome of treatment depend on early detection. Diagnosis is currently based on the detection of symptoms and neuroimaging abnormalities, which appear at relatively late stages in the pathogenesis. However, the underlying molecular responses to genetic and environmental insults begin much earlier and non-coding RNA networks are critically involved in these cellular regulatory mechanisms. Profiling RNA expression patterns could thus facilitate presymptomatic disease detection. Obtaining indirect readouts of pathological processes is particularly important for brain disorders because of the lack of direct access to tissue for molecular analyses. Living neurons and other CNS cells secrete microRNA and other small non-coding RNA into the extracellular space packaged in exosomes, microvesicles, or lipoprotein complexes. This discovery, together with the rapidly evolving massive sequencing technologies that allow detection of virtually all RNA species from small amounts of biological material, has allowed significant progress in the use of extracellular RNA as a biomarker for CNS malignancies, neurological, and psychiatric diseases. There is also recent evidence that the interactions between external stimuli and brain pathological processes may be reflected in peripheral tissues, facilitating their use as potential diagnostic markers. In this review, we explore the possibilities and challenges of using microRNA and other small RNAs as a signature for neurodegenerative and other neuropsychatric conditions.

## Introduction

Central nervous system disorders encompass a broad spectrum of neurodegenerative, oncological, inflammatory, and developmental conditions. Several mechanisms exist that evolved in order to isolate and protect the CNS from insult; interestingly, these effectively also act as barriers to diagnosis. Surrogate markers of disease are thus critical to facilitate disease detection, stratification of patients into subpopulations, prediction of prognosis, evaluation of response to treatment, and eventually allow better understanding of etiopathology.

To be of maximum diagnostic benefit, biomarkers would predict disease early, before the onset of clinical symptoms. Finding and testing such biomarkers would be best achieved by a longitudinal study in a large patient population at risk of developing the disease, a resource-intensive process that requires a long commitment and careful planning. However, the more common cross-sectional association studies are equally valuable in biomarker discovery. Brain imaging techniques and their modifications, as well as genotype studies to identify susceptibility alleles—the latter frequently employed in predicting tumor prognosis—are being used successfully to understand complex neurological conditions. In parallel, as techniques evolve rapidly and new hypotheses emerge, we see novel methods being applied to biomarker discovery. Thus, with the recent rapid acceleration in the field of non-coding RNA research, the potential predictive and diagnostic uses of these molecules have also attracted significant attention. Among non-coding RNA, microRNAs have been most intensely studied and their biology has repeatedly been proven critical for diverse cellular functions. More importantly, recent evidence indicates that miRNAs can be detected in peripheral tissues and can be used to “capture” changes in the cell of origin, including neurons. This has generated substantial interest in the use of small non-coding RNAs, in particular miRNAs, as biomarkers for CNS pathology. One advantage of molecular markers such as small RNAs over imaging technology is that samples can be frozen down for retrospective analysis, which enables larger studies. This manuscript aims to provide an overview of recent advances in the field of miRNA-based biomarker discovery for CNS disease.

## Sources of RNA biomarkers

**Figure 1:**
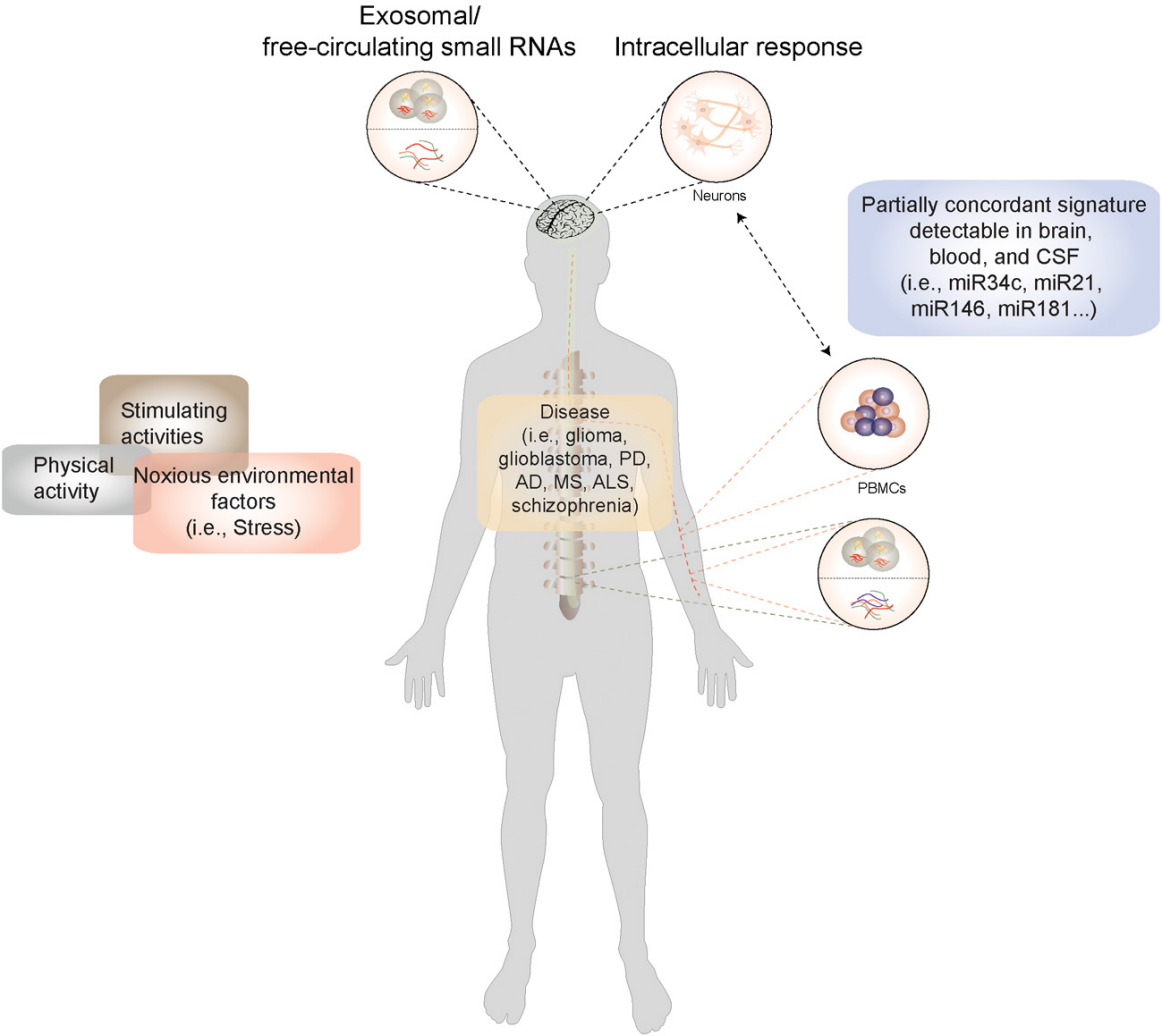
**A model for miRNA-based biomarker development: Disease-causing factors impact the brain both directly and indirectly (via immune and other cells), eliciting changes in gene and microRNA expression patterns**. Many of these stimuli concurrently exert their influence on non-neuronal cells, where they also elicit a response. In CNS diseases, in the absence of direct access to diseased tissue, microRNA expression patterns from peripheral cells such as blood cells could be used a proxy for genome-environment interaction in the CNS. Moreover, microRNAs circulate stably in cerebrospinal fluid and plasma in extracellular vesicles and in lipoprotein complexes, and can be isolated from these body fluids and profiled. Information derived from peripheral sources could thus be used to construct a picture of neuronal function both in the healthy and the diseased state.

As RNA is continually transcribed, translated, and turned over in response to physiological and pathological stimuli, the RNA profile of a cell, interpreted appropriately, could serve as a reflection of its current functional state. Current technologies enable transcriptome analysis on an unprecedented scale. In the human CNS we often need to rely on extracranial or peripheral sources of RNA to obtain a live readout of the disease state. The choice of potential sources for representative RNA is wide and includes body fluids such as blood, plasma, or cerebrospinal fluid as well as non-neuronal tissue or cells such as lymphocytes (Figure 1). The question that arises when using non-neuronal tissue or body fluids as a source is: To what degree do they resemble biological processes in the brain, arguably the most unique of organs with a distinct composition and cellular milieu? Nevertheless, a biomarker is formally defined as a proxy that allows remote and early detection of a biological process (i.e., disease) regardless of its mechanistic role in the condition being diagnosed. In the ideal situation, it would also reflect the biology of the original tissue, thus providing insight into disease mechanism, and even serve as a potential therapeutic target. Two major sources of peripheral RNA exist, namely extracellular RNA and RNA within peripheral mononuclear blood cells (PBMCs). While the former is still beginning to be explored, for the latter evidence has accumulated to indicate that a certain correlation exists between the molecular events occurring in the brain and those that can be detected in blood cells (Figure 1).

### RNA from blood cells

The use of genetic material from blood cells to screen for biomarkers of neurological conditions has been used as early as 1975 (Issidorides et al., 1975). Peripheral blood mononuclear cells (PBMCs), one of the major cellular components of blood, are particularly interesting in the context of biomarkers due to their ability not only to respond to internal and external stimuli, but also to “store” the information at the epigenetic level (Tang et al., 2001; Gavin and Sharma, 2009, 2010). Studies in monozygotic twins have demonstrated that over time PBMCs accumulate differences at the DNA methylation and histone acetylation level (Fraga et al., 2005). Furthermore, PBMCs have been successfully used to characterize the disease biosignature in neuropsychiatric conditions such as schizophrenia and bipolar disorder (Tang et al., 2001; Segman et al., 2005; Tsuang et al., 2005; Bowden et al., 2006; Iga et al., 2006; Anderson et al., 2008). Several lines of evidence suggest that both brain and blood cells can respond to environmental stimuli and reflect this response at the epigenetic level in their genome and that this response is indeed to some extent concordant between both tissue types (Desjardins et al., 2008; van Heerden et al., 2009; Li et al., 2011; Ursini et al., 2011; Yuferov et al., 2011; Davies et al., 2012; Provencal et al., 2012). Firstly, gene expression profiles in PBMCs have revealed common patterns of transcriptional activity in blood and neurons (Sullivan et al., 2006). Thus, for example, DNA methyltransferases DNMT1 and DNMT3a have been found to be upregulated in both postmortem brain tissue and PBMCs from schizophrenia patients (Zhubi et al., 2009) and whole chromosome mRNA expression profiles were found to be partially consistent between blood and brain in Huntington's disease patients (Anderson et al., 2008). In mice, a model of early life stress (i.e., maternal separation) was shown to induce a concordant transcriptional response in PBMCs and several brain regions (Desjardins et al., 2008).

Further, transcriptomic information obtained in peripheral blood has been successfully applied to predict healthy/disease status or to differentiate between disease stages (Tang et al., 2001; Tsuang et al., 2005; Du et al., 2006; Desjardins et al., 2008). This is possible due to the fact that PBMCs and neurons are actually exposed to very similar biochemical environments and can thus mount a concordant cellular response to incoming stimuli. Interestingly, in most of these cases, genes found to be differentially expressed in blood were also directly associated with neuropsychiatric disease and to be altered in postmortem brain (Tang et al., 2001; Tsuang et al., 2005; Du et al., 2006; Desjardins et al., 2008).

Secondly, the levels of certain epigenetic markers, such as DNA methylation patterns or miRNA expression, have been shown to directly correlate between PBMCs and neuronal tissue. A recent study by Davies and colleagues demonstrated a globally correlated inter-individual pattern of DNA methylation between cortical brain areas and PBMCs in healthy human postmortem tissue (Davies et al., 2012). In Rhesus monkeys, a model of early life stress based on surrogate mother rearing induced significant changes in DNA methylation in the prefrontal cortex, as well as in PBMCs (Provencal et al., 2012). Although the response in brain was more drastic, a positive and significant correlation in epigenetic changes was found between both tissue types (Provencal et al., 2012). At the individual gene level, the prodynorphin promoter has also been recently shown to display a consistent methylation pattern between blood cells and caudate/cingulate cortex in human post-mortem tissue (Ursini et al., 2011) and changes in methylation observed in human blood samples within the COMT gene (Catechol-O-methyltransferase, a critical enzyme for dopamine processing in the brain) were replicated and significantly correlated between blood and prefrontal cortex in the orthologous genomic location in rats (Li et al., 2011). Additionally, there is evidence to suggest that the level of other epigenetic markers, such as miRNA levels, also show parallel patterns of expression in blood and brain. Thus, levels of miR34a were recently shown to increase during aging in blood PBMCs, as well as in plasma and brain, and to correlate with a concomitant decrease in SIRT1 expression, one of the main targets of this miRNA (van Heerden et al., 2009).

Taken together, there is a solid base to suggest that PBMCs and perhaps other blood cells have the potential to provide a transcriptional and epigenetic biosignature that can be useful for both biomarker development and drug discovery and that these can be used as a proxy to study epigenetic mechanisms of neuropathology and its progression.

### Extracellular RNA

After the discovery that cells export RNA packaged in 40–90 nm sized vesicles called exosomes, and that this RNA could be taken up and translated by recipient cells (Valadi et al., 2007), extracellular vesicles rapidly attracted attention as a potential medium for intercellular communication. Similar findings in exosomes from primary glioblastoma cells, indicating that malignant vesicles may play a role in modulating tumor microenvironment (Skog et al., 2008), brought researchers to the idea of using the information carried by these vesicles to study organs/tumors remotely. Cell-derived RNA can also be found in a host of other membrane enclosed vesicular bodies variously called nanovesicles (Kogure et al., 2011), shedding vesicles, microvesicles (Ratajczak et al., 2006), or microparticles (Patz et al., 2013).

Exosomal and other extracellular vesicles are known to play a role in neuronal function, but the nature and degree of their involvement is still being studied. Exosomal release is modulated by glutamatergic synaptic activity, indicating that this may be a part of normal synapse physiology, and that the contents of these vesicles could be relevant for interneuronal communication (Lachenal et al., 2011). Exosomes also play a role in signaling between the pre- and post-synapse. Exosomal transfer of synaptotagmin 4 from the pre- to the post-synaptic compartment enables the presynapse to influence postsynaptic retrograde signaling (Korkut et al., 2013). These and several other lines of evidence led to the hypothesis that intercellular communication via exosomal content is a key underexplored physiological mechanism in the nervous system (Smalheiser, 2007). Thus, the RNA content of brain-cell-derived vesicles is a promising source of biomarkers for CNS disease. Extracellular RNA can also be found outside vesicles (Wang et al., 2010), in complex with lipoproteins such as HDL (Vickers et al., 2011) or with Argonaute2 (Arroyo et al., 2011; Turchinovich et al., 2011). This population comprises primarily miRNA, which appears to circulate stably in this form (Mitchell et al., 2008).

Recently, evidence that extracellular RNA can be extracted from various body fluids including saliva (Palanisamy et al., 2010), plasma (Hunter et al., 2008), urine (Alvarez et al., 2012), and CSF (Patz et al., 2013) has accumulated (Figure 1). Next generation sequencing (NGS)-generated profiles of the RNA contents of extracellular vesicles are beginning to be published (Burgos et al., 2013; Ogawa et al., 2013). However, the cellular source of this RNA is not always clear. RNA isolated from body fluids is likely to originate from a heterogenous mixture of cell types. The majority of RNA that circulates in the plasma is presumably of hematologic or endothelial cell origin, and the degree to which other tissues contribute is difficult to estimate. Studying the degree of variation of circulating miRNA molecules from the canonical sequence (the so-called isomiR profile) could allow an estimation of relative contributions of its tissue of origin (Williams et al., 2013). Although CSF is a relatively closed system, the cellular subpopulation of origin of CSF vesicles is also heterogenous, comprising vesicles derived from oligodendrocytes (Scolding et al., 1989), microglia, and macrophages (Verderio et al., 2012) as well as neurons (Saman et al., 2012).

Rapid progress is currently being made in the relatively new field of extracellular RNA isolation and profiling. Body fluids such as blood or CSF are thus likely to be a rich future source of small RNA biomarkers for CNS disease (Figure 1).

## Current microRNA detection and analysis technologies

CNS biomarker studies have employed RNA from several different sources, and the decision about choice of source RNA involves several factors. Using whole blood, serum, or plasma is clearly a minimally invasive approach and for those trying to develop or test a biomarker, these samples are probably easiest to access from registries or biological material repositories. Moreover, for ultimate clinical use, an accurate blood-based biomarker would be highly valuable. On the other hand, the presence of the blood-CSF barrier makes it likely that molecular entities isolated directly from CSF are more accurate reflections of brain physiological and pathological processes. Thus, RNA from CSF could be a more sensitive marker of changes that are diluted when trying to detect them in peripheral tissue. Using non-coding RNA as a molecular marker for disease involves several steps: The RNA must be isolated from the source and purified, enriched, or amplified before it is quantified, analyzed, and connected back to biological function. At each step of the process a formidable array of alternatives exists, and technologies in this field continue to evolve rapidly.

### Extracellular RNA isolation methods

RNA can be extracted from extracellular vesicles with relative ease, using one of several methods. The most commonly used isolation methods employ commercial kits based on a combination of a lysis step and column precipitation. Guanidinium thiocyanate-phenol-chloroform extraction is also effective, either by itself or in combination with a column. Most methods result in high quality and pure RNA, equally compatible with most downstream applications. However, each method results in a different RNA yield, in terms of quantity as well as RNA size profile (Eldh et al., 2012). One possible reason for that is that all the current vesicular isolation methods yield a heterogenous mixture of vesicles that vary in intracellular source (cell membrane vs. endosomal), RNA content, and lipid membrane composition. The difference in membrane composition likely translates to a difference in susceptibility to lysis, as different buffers are likely to target vesicle subpopulations with varying degrees of efficacy. Moreover, some of the commercially available methods are specifically designed to enrich small RNA species, while others are non-selective. The outcome is that the RNA population used for biomarker studies depends heavily on the RNA extraction method employed.

These differences in isolated RNA species are even wider when RNA is isolated directly from serum, plasma, CSF, or other biological fluids. The miRNA content is likely to include protein and lipid-complex associated free RNA in addition to vesicular RNA. A comparison of RNA extraction methods used directly on plasma and CSF showed large differences in yield (Burgos et al., 2013). The degree of variation in RNA size profile and content is not clear.

RNA can also be isolated from whole blood using commercially available tubes designed for the purpose. A comparison of 2 commercial kits using proprietary lysis reagents for direct RNA isolation from peripheral blood found that the overlap between the results obtained (in terms of gene expression changes) could be as low as 46% (Menke et al., 2012); this effect is particularly pronounced when the fold change in gene expression is small (Asare et al., 2008).

### miRNA detection/quantification

One step in miRNA detection is the sensitivity and accuracy of the technologies employed in their detection. In the case of small RNAs, there is a number of methods, from classical Northern Blotting to microarrays (Cissell and Deo, 2009; de Planell-Saguer and Rodicio, 2011). But if there is one technology that has allowed the leap in this field, it has been NGS. Although there has been great development in the techniques for small RNA detection and quantification, it was really the implementation of small RNA sequencing (small RNASeq) that made the difference in our knowledge of these molecules. In fact, the number of novel miRNAs has started growing exponentially since the implementation of small RNASeq sequencing (http://www.dddmag.com/articles/2012/12/starting-small). Techniques previously used to probe the cellular small RNAome are diverse and each of them has unique advantages and disadvantages to it, mainly associated with (1) whether detection is done in solid state or in solution and (2) whether or not previous knowledge of the target molecules is required [reviewed in Cissell and Deo (2009), de Planell-Saguer and Rodicio (2011)]. Briefly, solid-based technologies are more amenable to high-throughput strategies but are generally more time-consuming and have a difficult application *in vivo*, whereas solution-based techniques give much faster output and can be used *in vivo* but miss the global picture (Cissell and Deo, 2009; de Planell-Saguer and Rodicio, 2011). But arguably the currently hottest technique used for small RNA detection is small RNA sequencing. In this approach, total RNA is extracted and a size selection step ensures enrichment for small RNAs (18–22 nt in size). After adapter ligation, these are then subjected to sequencing, resulting in millions of reads that represent the abundance of each small RNA/miRNA molecule in the sample [although the degree of correlation between the actual abundance and read count is not free of debate (Linsen et al., 2009)]. This approach expands the dynamic range of signal for small RNA detection massively and provides unbiased interrogation of all known and unknown small RNA species without prior knowledge of the target, thereby virtually overcoming the limitations of all the other available technologies. If anything, one of the major limitations for the end-user of small RNASeq is the analysis (see following section).

As sequencing technologies continue to evolve rapidly while becoming more and more accessible to researchers, this method has taken over by far as the golden standard for small RNA expression analysis and novel discovery. It has been successfully used to model brain development (Yao et al., 2012), to characterize different mammalian tissues (Landgraf et al., 2007), and to study and develop biomarkers for different kinds of cancer (Moore et al., 2013), to name a few examples. Furthermore, one of the earliest studies to apply genome-wide small RNA profiling in neurons lead to the discovery of miR34c as a potential biomarker and a therapeutic target for Alzheimers's disease (Zovoilis et al., 2011). Additionally, because sequencing does not depend on previous target knowledge, there are more and more studies uncovering novel miRNAs and other small RNA species in the brain (Jacquier, 2009; Lee et al., 2009; Ling et al., 2011; Inukai et al., 2012). Naturally, sequencing-based approaches do entail some limitations. In addition to the still relatively complex analysis, the major disadvantages relate mainly to scalability and input material requirements. One of the steps in sample preparation is PCR amplification. It is a well-known source of biases and, if overdone, can cause excessive duplication levels, which leads to information loss during the analysis. Although the amount of input material is generally not problematic in most model system approaches, when dealing with human tissue, and, in particular, in the field of biomarker development, where sample access is limited (i.e., in the case of blood or cerebrospinal fluid), the ability to scale down starting material requirements is critical. The field of small RNASeq is still under heavy development and there is reason to believe that downscaling can indeed be achieved with high fidelity, at least pertaining to miRNA detection (authors' unpublished data). As sequencing technologies continue to develop, we will be able to detect small RNAs from very low amounts of starting biological material.

### Data analysis and pattern discovery

RNA-Seq data analysis entails serial steps including quality control, alignment to reference genome, read quantification (read counting), and statistical comparison of conditions of interests (Pepke et al., 2009). A comprehensive review of the method is out of the scope of this article, but it is worth mentioning that in the case of small RNAs, there are some additional considerations to be made. Because of the short length of target molecules, sequencers will read into the adapter primers used during the library preparation. These sequences have to be trimmed before alignment, since they would otherwise interfere with this step. The alignment step itself is also distinctive from the approach generally taken for RNA-Seq. Although alignment to the genome is possible, most current strategies take a hierarchical approach in which reads are serially aligned to different databases of small RNA species. After alignment, read counting and differential expression analysis can be carried out using standard procedures as those used in RNASeq (Pepke et al., 2009). Although the analytical procedure for small RNASeq is still under development, a number of publicly available tools exist that deal with the most standard approaches [the pros and cons of some of which are reviewed in Zhou et al. (2011)].

As small RNA studies evolve from investigation of single candidates to global transcriptional profiling, novel methods of analysis need to be adopted to interpret the large amounts of data generated. When targeted approaches are used, investigators typically use *p*-values or *p*-values corrected for multiple testing. With larger datasets, where differential expression analysis is the norm, filtering, and normalization is often of critical importance. These data also lend themselves very well to machine learning approaches, which have already been used in miRNA biomarker studies for multiple sclerosis and glioblastoma (Roth et al., 2011; Noerholm et al., 2012).

In biomarker research, the most commonly used unsupervised learning approaches are clustering and principle component analysis (PCA), typically used to detect a feature pattern without prior knowledge about sample grouping. In situations where the RNA profiles of the groups under comparison exhibit a high level of dissimilarity, they cluster into distinct groups by an unsupervised clustering algorithm. Alternatively, a “modified unsupervised clustering” where clustering is performed after feature selection may also be used (Noerholm et al., 2012). In most studies, the differences in RNA expression profiles are often subtle, requiring selection of candidates followed by application of supervised machine learning algorithms. Optimally applied, supervised machine learning algorithms such as support vector machines (the most popular so far in RNA biomarker studies), random forests, or artificial neural networks are trained to make classifications based on selected features and then tested on an independent data set to estimate prediction accuracy. However, flawed application of these specialized analysis techniques can lead to reporting of falsely high accuracy rates, hindering reproducibility.

For biomarkers to be used in the clinical setting, they should be applicable (with a certain margin of error) to a single individual. Therefore, predictions of sensitivity, specificity, and accuracy are often more useful than estimates of significant differences between patient and control groups.

## Landmark CNS biomarker work

Blood cells, plasma, and CSF have all been used as starting material to develop miRNA biomarkers for CNS malignancies as well as neurodegenerative and other neurological diseases. One of the first studies to compare miRNA profiles from blood mononuclear cells between patient and control populations showed mir-34a and mir 181b to be upregulated in mononuclear cells from the blood of patients with Alzheimer's disease. In addition, gender and APOE4 status were also found to influence the PBMC miRNA profiles within the group of AD patients (Schipper et al., 2007). This approach has since been used to identify potential biomarkers for other CNS diseases such as multiple sclerosis, schizophrenia (Lai et al., 2011; Gardiner et al., 2012), Parkinson's disease (Martins et al., 2011; Soreq et al., 2013), and amyotrophic lateral sclerosis (De Felice et al., 2012). For multiple sclerosis in particular, a large number of studies exist that profile miRNA in peripheral blood immune cells (Keller et al., 2009; Cox et al., 2010; De Santis et al., 2010; Lindberg et al., 2010; Martinelli-Boneschi et al., 2012).

Plasma and serum have also been investigated as a source of miRNA biomarkers for multiple sclerosis (Siegel et al., 2012). Cerebrospinal fluid miRNA has been studied in Alzheimer's disease (Cogswell et al., 2008), multiple sclerosis (Haghikia et al., 2012), and to a larger extent in glioblastoma (Baraniskin et al., 2012; Teplyuk et al., 2012). A single study of miRNA in pooled CSF microparticles from patients with neurotrauma showed that the contents of CSF could also be useful in diagnosing brain injury (Patz et al., 2013) (Table 1). Among the CNS malignancies, a variety of starting biological materials has been used; the majority of studies investigate samples from patients with glioblastoma, probably because drawing CSF pre and post-operatively is routine procedure in glioblastoma diagnosis. (Roth et al., 2011; Baraniskin et al., 2012; Ilhan-Mutlu et al., 2012; Teplyuk et al., 2012; Wang et al., 2012), and a single study of patients with astrocytoma (Yang et al., 2013) (Table 1).

**Table 1:**
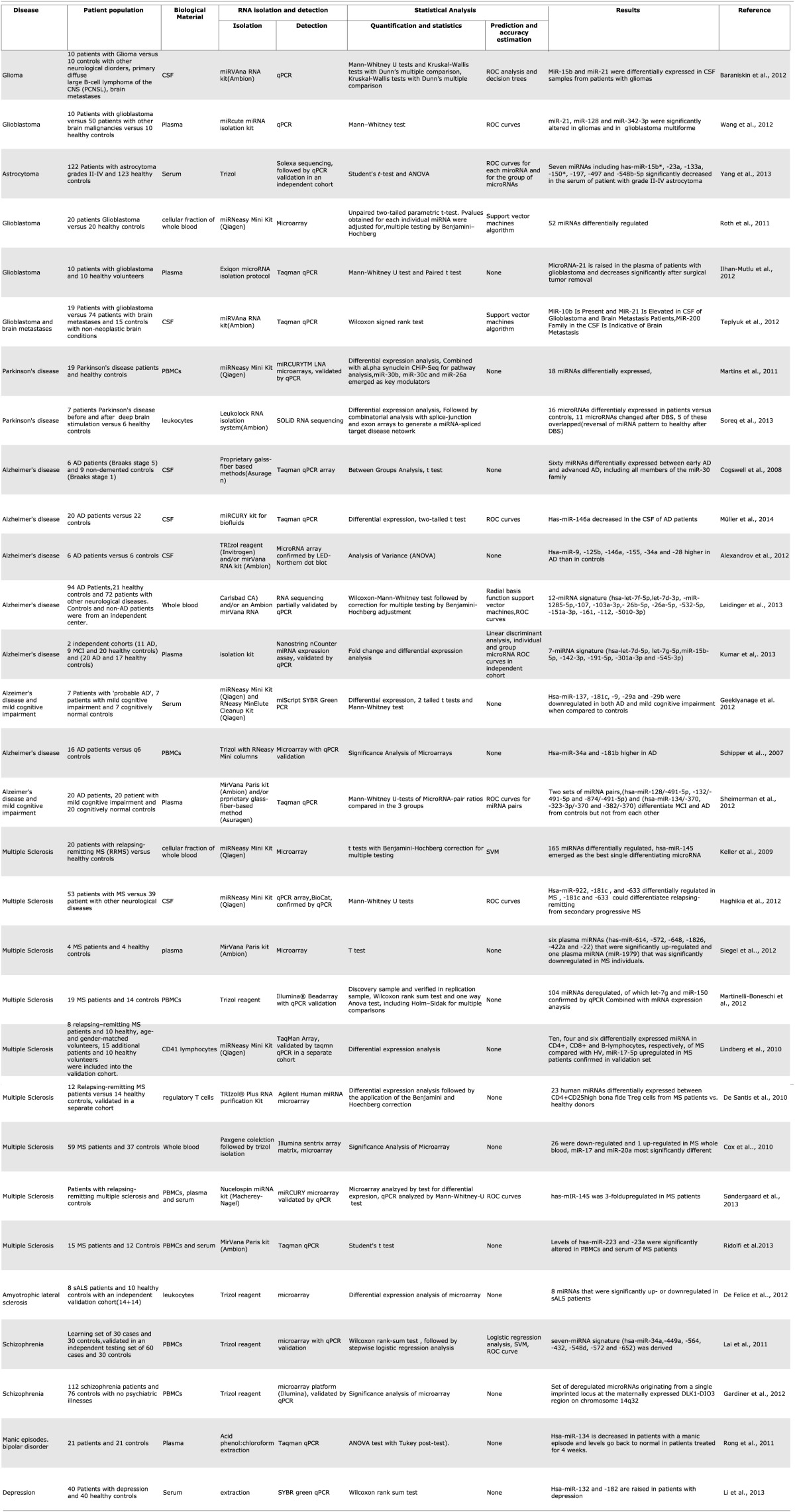
**Summary of microRNA biomarker studies for central nervous system diseases**.

Over the last year there has been a sharp increase in published studies about circulating microRNA as biomarkers for various neurological diseases. Many of these used unbiased, genome-wide profiling approaches to compare patients with controls and derive. For Alzheimer's disease alone there are now a total of 5 published studies from various blood fractions and 3 from CSF. While these individual studies report high accuracy rates, and some of them include large numbers of patients, it is curious that their results do not match or even overlap with each other. The blood studies all used different fractions of blood and comparisons are perhaps unrealistic, but the CSF studies also showed differing results. For example, hsa-miR-146a is reported in one of the 3 studies to be upregulated in AD (Alexandrov et al., 2012), in a second study to be downregulated (Müller et al., 2014), while the third shows no effect on it at all, reporting a downregulation of hsa-miR-146b instead (Cogswell et al., 2008) (Table 1). Perhaps in the future, a larger number of studies and their metaanalysis would shed more light on which non-coding RNAs are truly useful biomarkers of disease.

## From biomarkers to function

Although several classes of non-coding RNA have been discovered (Taft et al., 2010), miRNAs are the most extensively characterized. Computational tools that predict miRNA targets are quite frequently used to ascribe function to putative miRNA biomarkers. Since miRNAs and the genes they target are expressed in a tissue- and pathology-specific manner, predicted targets usually require experimental confirmation. Tools that combine prediction algorithms with large scale wet lab experimental methods such as polysome profiling, immunoprecipitation of members of the RISC complex or degradome sequencing are likely to provide more specific results (Thomson et al., 2011). Since the publication of a miRNA mRNA map based on argonaute HITS-CLIP data from the brain (Chi et al., 2009), more specific predictions are also available.

As our understanding of non-coding RNA biology develops, we see that miRNAs are evolutionarily conserved across species but have overlapping targets and are often functionally redundant. While landmark advances have been made toward understanding the role of single miRNAs in the CNS (Kim et al., 2007; Rajasethupathy et al., 2009; Edbauer et al., 2010; Zovoilis et al., 2011), we see a gradual shift from studying the single-miRNA-target interaction toward viewing these critical regulators as part of a network, tuning or buffering key gene regulation node (Zhang and Su, 2009).

Clearly, miRNAs exert their influence on biological pathways in concert with transcription factors and other modulators of gene expression. A few of the more recent biomarker studies attempt to view the larger picture by concurrently profiling miRNA expression, gene expression, and protein-DNA interaction. In particular, researchers studying biomarkers for Parkinson's disease have pioneered these analyses by combining miRNA expression with tissue-specific gene isoform expression (Soreq et al., 2013) or data from ChIP-sequencing data with miRNA target prediction (Martins et al., 2011) to build a picture of the regulatory network in health vs. disease.

Biomarkers are ultimately validated when they can be connected with molecular mechanisms across different levels of biological complexity. A systems biology approach could achieve this by integrating data, where it is available, across different levels such as genes, molecules, phenotypes, cell, and tissues. Various computational tools are available to integrate these data types and more are being developed (Villoslada and Baranzini, 2012). Simple, readily available and widely used methods to link a set of differentially expressed genes with biological processes or pathways include gene ontology term search and *gene set enrichment analysis*. The availability of large and complex data sets and computing power has spurred rapid advances in network biology.

Moreover, RNA data can be analyzed in combination with patient information, disease history, genomic data like APOE4 allele, disease-specific clinical tests like MEP (motor-evoked potential for MS or mini-mental state examination for dementia), and data from proteomics and other high throughput approaches. Proteomics-based biomarkers for neurodegenerative and other neurological diseases have been studied and new avenues for biomarker discovery such as metabolomics continue to emerge; an LC/MS based approach (Trushina et al., 2013) to study the metabolic profiles of CSF and plasma from AD patients found around 150 metabolites each in CSF and plasma that were significantly different in patients with Alzheimer's disease or patients with mild cognitive impairment (MCI) than healthy individuals, allowing them to identify putative pathways that may be altered (Trushina et al., 2013). These kinds of data could lend themselves to a combinatorial analysis provided that patient information and other variables are fully documented and available.

## Current limitations and future milestones of miRNA-based biomarker discovery

The use of non-coding RNA and miRNA in particular has gained significant attention since the discovery that these RNA species can be detected extra- and intracellularly in peripheral tissue. The growing use of powerful detection methods such as massive sequencing has given a significant boost to the search for minimally invasive disease indicator. In addition, the discovery of the existence of free or exosomal circulating RNA in blood and CSF has also fostered research in this direction. Although this is still a relatively young field, it is rapidly evolving and promises great advances in the field of biomarker discovery, especially for nervous system pathology. The CNS is the least accessible of all tissues and would therefore greatly benefit from advances in this field. Current limitations to this approach include those inherently associated with biomarker discovery (i.e., working with material from different sources, extraction methods, patient history, etc.), as well as those specifically associated with sequencing-based detection methods and extraction strategies.

As is often the case when working with human tissue, samples from different sources show wide variability in profile as a result of handling, sample preparation and preservation. These are especially pronounced when a highly sensitive technique like sequencing is used. In addition, because the source of tissue are primarily human patients that may be on medication, proper consideration of these (potentially confounding) cofactors is essential, as medication pursues restoration of the biological balance and this may include alterations in the molecule of interest. When RNA profiles are altered after drug treatment, it can be a challenge to dissect the direct effects of treatment on RNA expression from those connected with disease remission (Rong et al., 2011). An analysis of highly cited (more than 400 citations) biomarker publications (including protein, genetic, and other blood biomarkers) showed that individual studies usually report high association between the marker and disease outcome; however when the same biomarker is subsequently compared with larger studies or metaanalyses, the effect size is often significantly smaller than initially believed (Ioannidis and Panagiotou, 2011).

Another issue inherently associated with large human studies and generally with studies handling big datasets is information availability and reproducibility. As is known from the field of microarrays, data is often incomplete or incompletely annotated and the analyses hard to reproduce (Ioannidis et al., 2009) and this is still an issue in the field of small RNA-based biomarker development (Ioannidis et al., 2009).

In addition to these limitations, there is also those specifically associated with the extraction and quantification methods used for peripheral miRNA detection. As already mentioned in section Current microRNA Detection and Analysis Technologies, a variety of extraction techniques exist, each with specific biases that can greatly influence the relative weight of a certain molecular species in the sample. In addition, because the technology is rapidly evolving, there is still no clear-cut consensus as to what is the best approach to analyze large-scale small RNA profiles. These issues will settle with time, as techniques become more robust and analysis methods stabilize, but until then, they are to be carefully considered in the experimental design.

Finally, as already mentioned, there is the issue of how faithful the peripheral profile is to the original biological situation in the CNS. Although this is not most critical for biomarker discovery *per se* (as mentioned above, a biomarker can be simply defined as a “handle” that allows detection of a remote biological process and does not necessarily need to correlate with it), often studies strive to uncover molecules that can serve as a biomarker *and* be used as therapeutic targets. Evidence from PBMCs indicates that there is indeed a considerable coherence between the central neuronal response and the peripheral response in blood and that there is a cross-talk between these two tissues. It remains to be experimentally established whether this correlation can also serve to better understand neuronal physiology in the healthy and the disease situation. In this respect, the development of novel, unbiased technologies to detect even the smallest amounts of miRNAs peripherally in combination with studies in model systems has proven critical.

All in all, despite current limitations, miRNA-based biomarkers constitute an exciting field in biomedical research. For neuroscience, where the search for remotely accessible markers to understand the brain is essential for human studies, the field has elicited considerable interest and as the costs of NGS continue to decrease, it is likely to become a routine approach to generate individual patient profiles and allow targeted therapeutic intervention.

### Conflict of interest statement

The authors declare that the research was conducted in the absence of any commercial or financial relationships that could be construed as a potential conflict of interest.

## References

[B1] Alexandrov P. N. Dua P. Hill J. M. Bhattacharjee S. Zhao Y. Lukiw W. J. (2012). microRNA (miRNA) speciation in Alzheimer's disease (AD) cerebrospinal fluid (CSF) and extracellular fluid (ECF). Int. J. Biochem. Mol. Biol. 3, 365–373 Available online at: http://www.ijbmb.org/files/ijbmb1211001.pdf 23301201 PMC3533883

[B2] Alvarez M. Khosroheidari M. Kanchi Ravi R. DiStefano J. (2012). Comparison of protein, microRNA, and mRNA yields using different methods of urinary exosome isolation for the discovery of kidney disease biomarkers. Kidney Int. 82, 1024–1032 10.1038/ki.2012.256 22785172

[B3] Anderson A. N. Roncaroli F. Hodges A. Deprez M. Turkheimer F. E. (2008). Chromosomal profiles of gene expression in Huntington's disease. Brain 131(Pt 2), 381–388 10.1093/brain/awm312 18156153

[B4] Arroyo J. Chevillet J. Kroh E. Ruf I. Pritchard C. Gibson D. (2011). Argonaute2 complexes carry a population of circulating microRNAs independent of vesicles in human plasma. Proc. Natl. Acad. Sci. U.S.A. 108, 5003–5008 10.1073/pnas.1019055108 21383194 PMC3064324

[B5] Asare A. Kolchinsky S. Gao Z. Wang R. Raddassi K. Bourcier K. (2008). Differential gene expression profiles are dependent upon method of peripheral blood collection and RNA isolation. BMC Genomics 9:474 10.1186/1471-2164-9-474 18847473 PMC2573897

[B6] Baraniskin A. Kuhnhenn J. Schlegel U. Maghnouj A. Zöllner H. Schmiegel W. (2012). Identification of microRNAs in the cerebrospinal fluid as biomarker for the diagnosis of glioma. Neuro Oncol. 14, 29–33 10.1093/neuonc/nor169 21937590 PMC3245991

[B7] Bowden N. A. Weidenhofer J. Scott R. J. Schall U. Todd J. Michie P. T. (2006). Preliminary investigation of gene expression profiles in peripheral blood lymphocytes in schizophrenia. Schizophr. Res. 82, 175–183 10.1016/j.schres.2005.11.012 16414245

[B8] Burgos K. Javaherian A. Bomprezzi R. Ghaffari L. Rhodes S. Courtright A. (2013). Identification of extracellular miRNA in human cerebrospinal fluid by next-generation sequencing. RNA 19, 712–722 10.1261/rna.036863.112 23525801 PMC3677285

[B9] Chi S. W. Zang J. B. Mele A. Darnell R. B. (2009). Argonaute HITS-CLIP decodes microRNA-mRNA interaction maps. Nature 460, 479–486 10.1038/nature08170 19536157 PMC2733940

[B10] Cissell K. A. Deo S. K. (2009). Trends in microRNA detection. Anal. Bioanal. Chem. 394, 1109–1116 10.1007/s00216-009-2744-6 19367400

[B11] Cogswell J. Ward J. Taylor I. Waters M. Shi Y. Cannon B. (2008). Identification of miRNA changes in Alzheimer's disease brain and CSF yields putative biomarkers and insights into disease pathways. J. Alzheimers Dis. 14, 27–41 Available online at: http://iospress.metapress.com/content/gw2umx21n3811184 18525125 10.3233/jad-2008-14103

[B12] Cox M. Cairns M. Gandhi K. Carroll A. Moscovis S. Stewart G. (2010). MicroRNAs miR-17 and miR-20a inhibit T cell activation genes and are under-expressed in MS whole blood. PLoS ONE 5:e12132 10.1371/journal.pone.0012132 20711463 PMC2920328

[B13] Davies M. N. Volta M. Pidsley R. Lunnon K. Dixit A. Lovestone S. (2012). Functional annotation of the human brain methylome identifies tissue-specific epigenetic variation across brain and blood. Genome Biol. 13, R43 10.1186/gb-2012-13-6-r43 22703893 PMC3446315

[B14] De Felice B. Guida M. Guida M. Coppola C. De Mieri G. Cotrufo R. (2012). A miRNA signature in leukocytes from sporadic amyotrophic lateral sclerosis. Gene 508, 35–40 10.1016/j.gene.2012.07.058 22903028

[B15] de Planell-Saguer M. Rodicio M. C. (2011). Analytical aspects of microRNA in diagnostics: a review. Anal. Chim. Acta 699, 134–152 10.1016/j.aca.2011.05.025 21704768

[B16] De Santis G. Ferracin M. Biondani A. Caniatti L. Rosaria Tola M. Castellazzi M. (2010). Altered miRNA expression in T regulatory cells in course of multiple sclerosis. J. Neuroimmunol. 226, 165–171 10.1016/j.jneuroim.2010.06.009 20637509

[B17] Desjardins S. Belkai E. Crete D. Cordonnier L. Scherrmann J. M. Noble F. (2008). Effects of chronic morphine and morphine withdrawal on gene expression in rat peripheral blood mononuclear cells. Neuropharmacology 55, 1347–1354 10.1016/j.neuropharm.2008.08.027 18801381

[B18] Du X. Tang Y. Xu H. Lit L. Walker W. Ashwood P. (2006). Genomic profiles for human peripheral blood T cells, B cells, natural killer cells, monocytes, and polymorphonuclear cells: comparisons to ischemic stroke, migraine, and Tourette syndrome. Genomics 87, 693–703 10.1016/j.ygeno.2006.02.003 16546348

[B19] Edbauer D. Neilson J. Foster K. Wang C.-F. Seeburg D. Batterton M. (2010). Regulation of synaptic structure and function by FMRP-associated microRNAs miR-125b and miR-132. Neuron 65, 373–384 10.1016/j.neuron.2010.01.005 20159450 PMC5018398

[B20] Eldh M. Lötvall J. Malmhäll C. Ekström K. (2012). Importance of RNA isolation methods for analysis of exosomal RNA: evaluation of different methods. Mol. Immunol. 50, 278–286 10.1016/j.molimm.2012.02.001 22424315

[B21] Fraga M. F. Ballestar E. Paz M. F. Ropero S. Setien F. Ballestar M. L. (2005). Epigenetic differences arise during the lifetime of monozygotic twins. Proc. Natl. Acad. Sci. U.S.A. 102, 10604–10609 10.1073/pnas.0500398102 16009939 PMC1174919

[B22] Gardiner E. Beveridge N. Wu J. Carr V. Scott R. Tooney P. (2012). Imprinted DLK1-DIO3 region of 14q32 defines a schizophrenia-associated miRNA signature in peripheral blood mononuclear cells. Mol. Psychiatry 17, 827–840 10.1038/mp.2011.78 21727898 PMC3404364

[B23] Gavin D. P. Sharma R. P. (2009). Chromatin from peripheral blood mononuclear cells as biomarkers for epigenetic abnormalities in schizophrenia. Cardiovasc. Psychiatry Neurol. 2009:409562 10.1155/2009/409562 20029620 PMC2790150

[B24] Gavin D. P. Sharma R. P. (2010). Histone modifications, DNA methylation, and schizophrenia. Neurosci. Biobehav. Rev. 34, 882–888 10.1016/j.neubiorev.2009.10.010 19879893 PMC2848916

[B24a] Geekiyanage H. Jicha G. A. Nelson P. T. Chan C. (2012). Blood serum miRNA: non-invasive biomarkers for Alzheimer's disease. Exp. Neurol. 235, 491–496 10.1016/j.expneurol.2011.11.026 22155483 PMC3361462

[B25] Haghikia A. Haghikia A. Hellwig K. Baraniskin A. Holzmann A. Décard B. (2012). Regulated microRNAs in the CSF of patients with multiple sclerosis: a case-control study. Neurology 79, 2166–2170 10.1212/WNL.0b013e3182759621 23077021

[B26] Hunter M. Ismail N. Zhang X. Aguda B. Lee E. Yu L. (2008). Detection of microRNA expression in human peripheral blood microvesicles. PLoS ONE 3:e3694 10.1371/journal.pone.0003694 19002258 PMC2577891

[B27] Iga J. Ueno S. Yamauchi K. Numata S. Motoki I. Tayoshi S. (2006). Gene expression and association analysis of LIM (PDLIM5) in major depression. Neurosci. Lett. 400, 203–207 10.1016/j.neulet.2006.02.044 16595163

[B28] Ilhan-Mutlu A. Wagner L. Wöhrer A. Furtner J. Widhalm G. Marosi C. (2012). Plasma MicroRNA-21 concentration may be a useful biomarker in glioblastoma patients. Cancer Invest. 30, 615–621 10.3109/07357907.2012.708071 22891879

[B29] Inukai S. de Lencastre A. Turner M. Slack F. (2012). Novel microRNAs differentially expressed during aging in the mouse brain. PLoS ONE 7:e40028 10.1371/journal.pone.0040028 22844398 PMC3402511

[B30] Ioannidis J. Allison D. Ball C. Coulibaly I. Cui X. Culhane A. (2009). Repeatability of published microarray gene expression analyses. Nat. Genet. 41, 149–155 10.1038/ng.295 19174838

[B31] Ioannidis J. Panagiotou O. (2011). Comparison of effect sizes associated with biomarkers reported in highly cited individual articles and in subsequent meta-analyses. JAMA 305, 2200–2210 10.1001/jama.2011.713 21632484

[B32] Issidorides M. R. Stefanis C. N. Varsou E. Katsorchis T. (1975). Altered chromatin ultrastructure in neutrophils of schizophrenics. Nature 258, 612–614 10.1038/258612a0 1207736

[B33] Jacquier A. (2009). The complex eukaryotic transcriptome: unexpected pervasive transcription and novel small RNAs. Nat. Rev. Genet. 10, 833–844 10.1038/nrg2683 19920851

[B34] Keller A. Leidinger P. Lange J. Borries A. Schroers H. Scheffler M. (2009). Multiple sclerosis: microRNA expression profiles accurately differentiate patients with relapsing-remitting disease from healthy controls. PLoS ONE 4:e7440 10.1371/journal.pone.0007440 19823682 PMC2757919

[B35] Kim J. Inoue K. Ishii J. Vanti W. Voronov S. Murchison E. (2007). A MicroRNA feedback circuit in midbrain dopamine neurons. Science 317, 1220–1224 10.1126/science.1140481 17761882 PMC2782470

[B36] Kogure T. Lin W.-L. Yan I. Braconi C. Patel T. (2011). Intercellular nanovesicle-mediated microRNA transfer: a mechanism of environmental modulation of hepatocellular cancer cell growth. Hepatology 54, 1237–1248 10.1002/hep.24504 21721029 PMC3310362

[B37] Korkut C. Li Y. Koles K. Brewer C. Ashley J. Yoshihara M. (2013). Regulation of postsynaptic retrograde signaling by presynaptic exosome release. Neuron 77, 1039–1046 10.1016/j.neuron.2013.01.013 23522040 PMC3626103

[B37a] Kumar P. Dezso Z. Mackenzie C. Oestreicher J. Agoulnik S. Byrne M. (2013). Circulating miRNA biomarkers for Alzheimer's Disease. PLoS ONE 8:e69807 10.1371/journal.pone.0069807 23922807 PMC3726785

[B38] Lachenal G. Pernet-Gallay K. Chivet M. Hemming F. Belly A. Bodon G. (2011). Release of exosomes from differentiated neurons and its regulation by synaptic glutamatergic activity. Mol. Cell. Neurosci. 46, 409–418 10.1016/j.mcn.2010.11.004 21111824

[B39] Lai C.-Y. Yu S.-L. Hsieh M. Chen C.-H. Chen H.-Y. Wen C.-C. (2011). MicroRNA expression aberration as potential peripheral blood biomarkers for schizophrenia. PLoS ONE 6:e21635 10.1371/journal.pone.0021635 21738743 PMC3126851

[B40] Landgraf P. Rusu M. Sheridan R. Sewer A. Iovino N. Aravin A. (2007). A mammalian microRNA expression atlas based on small RNA library sequencing. Cell 129, 1401–1414 10.1016/j.cell.2007.04.040 17604727 PMC2681231

[B41] Lee Y. S. Shibata Y. Malhotra A. Dutta A. (2009). A novel class of small RNAs: tRNA-derived RNA fragments (tRFs). Genes Dev. 23, 2639–2649 10.1101/gad.1837609 19933153 PMC2779758

[B41a] Leidinger P. Backes C. Deutscher S. Schmitt K. Muller S. C. Frese K. (2013). A blood based 12-miRNA signature of Alzheimer disease patients. Genome Biol. 14, R78 10.1186/gb-2013-14-7-r78 23895045 PMC4053778

[B42] Li X. Khanna A. Li N. Wang E. (2011). Circulatory miR34a as an RNAbased, noninvasive biomarker for brain aging. Aging 3, 985–1002 Available online at: http://www.impactaging.com/papers/v3/n10/full/100371.html 22064828 10.18632/aging.100371 PMC3229974

[B42a] Li Y. J. Xu M. Gao Z. H. Wang Y. Q. Yue Z. Zhang Y. X. (2013). Alterations of serum levels of BDNF-related miRNAs in patients with depression. PLoS ONE 8:e63648 10.1371/journal.pone.0063648 23704927 PMC3660391

[B43] Lindberg R. Hoffmann F. Mehling M. Kuhle J. Kappos L. (2010). Altered expression of miR-17-15p in CD4+ lymphocytes of relapsing-remitting multiple sclerosis patients. Eur. J. Immunol. 40, 888–898 10.1002/eji.200940032 20148420

[B44] Ling K. H. Brautigan P. J. Hahn C. N. Daish T. Rayner J. R. Cheah P. S. (2011). Deep sequencing analysis of the developing mouse brain reveals a novel microRNA. BMC Genomics 12:176 10.1186/1471-2164-12-176 21466694 PMC3088569

[B45] Linsen S. E. de Wit E. Janssens G. Heater S. Chapman L. Parkin R. K. (2009). Limitations and possibilities of small RNA digital gene expression profiling. Nat. Methods 6, 474–476 10.1038/nmeth0709-474 19564845

[B46] Martinelli-Boneschi F. Fenoglio C. Brambilla P. Sorosina M. Giacalone G. Esposito F. (2012). MicroRNA and mRNA expression profile screening in multiple sclerosis patients to unravel novel pathogenic steps and identify potential biomarkers. Neurosci. Lett. 508, 4–8 10.1016/j.neulet.2011.11.006 22108567

[B47] Martins M. Rosa A. Guedes L. Fonseca B. Gotovac K. Violante S. (2011). Convergence of miRNA expression profiling, α-synuclein interacton and GWAS in Parkinson's disease. PLoS ONE 6:e25443 10.1371/journal.pone.0025443 22003392 PMC3189215

[B48] Menke A. Rex-Haffner M. Klengel T. Binder E. Mehta D. (2012). Peripheral blood gene expression: it all boils down to the RNA collection tubes. BMC Res. Notes 5:1 10.1186/1756-0500-5-1 22214347 PMC3280191

[B49] Mitchell P. Parkin R. Kroh E. Fritz B. Wyman S. Pogosova-Agadjanyan E. (2008). Circulating microRNAs as stable blood-based markers for cancer detection. Proc. Natl. Acad. Sci. U.S.A. 105, 10513–10518 10.1073/pnas.0804549105 18663219 PMC2492472

[B50] Moore L. M. Kivinen V. Liu Y. Annala M. Cogdell D. Liu X. (2013). Transcriptome and small RNA deep sequencing reveals deregulation of miRNA biogenesis in human glioma. J. Pathol. 229, 449–459 10.1002/path.4109 23007860 PMC3857031

[B51] Müller M. Kuiperij H. B. Claassen J. A. Kusters B. Verbeek M. M. (2014). MicroRNAs in Alzheimer's disease: differential expression in hippocampus and cell-free cerebrospinal fluid. Neurobiol. Aging 35, 152–158 10.1016/j.neurobiolaging.2013.07.005 23962497

[B52] Noerholm M. Balaj L. Limperg T. Salehi A. Zhu L. D. Hochberg F. H. (2012). RNA expression patterns in serum microvesicles from patients with glioblastoma multiforme and controls. BMC Cancer 12:22 10.1186/1471-2407-12-22 22251860 PMC3329625

[B53] Ogawa Y. Taketomi Y. Murakami M. Tsujimoto M. Yanoshita R. (2013). Small RNA transcriptomes of two types of exosomes in human whole saliva determined by next generation sequencing. Biol. Pharm. Bull. 36, 66–75 10.1248/bpb.b12-00607 23302638

[B54] Palanisamy V. Sharma S. Deshpande A. Zhou H. Gimzewski J. Wong D. (2010). Nanostructural and transcriptomic analyses of human saliva derived exosomes. PLoS ONE 5:e8577 10.1371/journal.pone.0008577 20052414 PMC2797607

[B55] Patz S. Trattnig C. Grunbacher G. Ebner B. Gully C. Novak A. (2013). More than cell dust: microparticles isolated from cerebrospinal fluid of brain injured patients are messengers carrying mRNAs, miRNAs, and proteins. J. Neurotrauma 30, 1232–1242 10.1089/neu.2012.2596 23360174

[B56] Pepke S. Wold B. Mortazavi A. (2009). Computation for ChIP-seq and RNA-seq studies. Nat. Methods 6(11 Suppl.), S22–S32 10.1038/nmeth.1371 19844228 PMC4121056

[B57] Provencal N. Suderman M. J. Guillemin C. Massart R. Ruggiero A. Wang D. (2012). The signature of maternal rearing in the methylome in rhesus macaque prefrontal cortex and T cells. J. Neurosci. 32, 15626–15642 10.1523/JNEUROSCI.1470-12.2012 23115197 PMC3490439

[B58] Rajasethupathy P. Fiumara F. Sheridan R. Betel D. Puthanveettil S. Russo J. (2009). Characterization of small RNAs in Aplysia reveals a role for miR-124 in constraining synaptic plasticity through CREB. Neuron 63, 803–817 10.1016/j.neuron.2009.05.029 19778509 PMC2875683

[B59] Ratajczak J. Miekus K. Kucia M. Zhang J. Reca R. Dvorak P. (2006). Embryonic stem cell-derived microvesicles reprogram hematopoietic progenitors: evidence for horizontal transfer of mRNA and protein delivery. Leukemia 20, 847–856 10.1038/sj.leu.2404132 16453000

[B59a] Ridolfi E. Fenoglio C. Cantoni C. Calvi A. De Riz M. Pietroboni A. (2013). Expression and genetic analysis of microRNAs involved in multiple sclerosis. Int. J. Mol. Sci. 14, 4375–4384 10.3390/ijms14034375 23439547 PMC3634436

[B60] Rong H. Liu T. Yang K. Yang H. Wu D. Liao C. (2011). MicroRNA-134 plasma levels before and after treatment for bipolar mania. J. Psychiatr. Res. 45, 92–95 10.1016/j.jpsychires.2010.04.028 20546789

[B61] Roth P. Wischhusen J. Happold C. Chandran P. Hofer S. Eisele G. (2011). A specific miRNA signature in the peripheral blood of glioblastoma patients. J. Neurochem. 118, 449–457 10.1111/j.1471-4159.2011.07307.x 21561454

[B62] Saman S. Kim W. Raya M. Visnick Y. Miro S. Saman S. (2012). Exosome-associated tau is secreted in tauopathy models and is selectively phosphorylated in cerebrospinal fluid in early Alzheimer disease. J. Biol. Chem. 287, 3842–3849 10.1074/jbc.M111.277061 22057275 PMC3281682

[B63] Schipper H. Maes O. Chertkow H. Wang E. (2007). MicroRNA expression in Alzheimer blood mononuclear cells. Gene Regul. Syst. Biol. 1, 263–274 Available online at: http://www.la-press.com/microrna-expression-in-alzheimer-blood-mononuclear-cells-article-a483 19936094 10.4137/grsb.s361 PMC2759133

[B64] Scolding N. Morgan B. Houston W. Linington C. Campbell A. Compston D. (1989). Vesicular removal by oligodendrocytes of membrane attack complexes formed by activated complement. Nature 339, 620–622 10.1038/339620a0 2733792

[B65] Segman R. H. Shefi N. Goltser-Dubner T. Friedman N. Kaminski N. Shalev A. Y. (2005). Peripheral blood mononuclear cell gene expression profiles identify emergent post-traumatic stress disorder among trauma survivors. Mol. Psychiatry 10, 500–513 425. 10.1038/sj.mp.4001636 15685253

[B65a] Sheinerman K. S. Tsivinsky V. G. Crawford F. Mullan M. J. Abdullah L. Umansky S. R. (2012). Plasma microRNA biomarkers for detection of mild cognitive impairment. Aging (Albany NY) 4, 590-605 23001356 10.18632/aging.100486 PMC3492224

[B66] Siegel S. Mackenzie J. Chaplin G. Jablonski N. Griffiths L. (2012). Circulating microRNAs involved in multiple sclerosis. Mol. Biol. Rep. 39, 6219–6225 10.1007/s11033-011-1441-7 22231906

[B67] Skog J. Würdinger T. van Rijn S. Meijer D. Gainche L. Sena-Esteves M. (2008). Glioblastoma microvesicles transport RNA and proteins that promote tumour growth and provide diagnostic biomarkers. Nat. Cell Biol. 10, 1470–1476 10.1038/ncb1800 19011622 PMC3423894

[B68] Smalheiser N. (2007). Exosomal transfer of proteins and RNAs at synapses in the nervous system. Biol. Dir. 2, 35 10.1186/1745-6150-2-35 18053135 PMC2219957

[B68a] Søndergaard H. B. Hesse D. Krakauer M. Sorensen P. S. Sellebjerg F. (2013). Differential microRNA expression in blood in multiple sclerosis. Mult. Scler. [Epub ahead of print]. 10.1177/1352458513490542 23773985

[B69] Soreq L. Salomonis N. Bronstein M. Greenberg D. Israel Z. Bergman H. (2013). Small RNA sequencing-microarray analyses in Parkinson leukocytes reveal deep brain stimulation-induced splicing changes that classify brain region transcriptomes. Front. Mol. Neurosci. 6:10 10.3389/fnmol.2013.00010 23717260 PMC3652308

[B70] Sullivan P. F. Fan C. Perou C. M. (2006). Evaluating the comparability of gene expression in blood and brain. Am. J. Med. Genet. B Neuropsychiatr. Genet. 141B, 261–268 10.1002/ajmg.b.30272 16526044

[B71] Taft R. J. Pang K. C. Mercer T. R. Dinger M. Mattick J. S. (2010). Non-coding RNAs: regulators of disease. J. Pathol. 220, 126–139 10.1002/path.2638 19882673

[B72] Tang Y. Lu A. Aronow B. J. Sharp F. R. (2001). Blood genomic responses differ after stroke, seizures, hypoglycemia, and hypoxia: blood genomic fingerprints of disease. Ann. Neurol. 50, 699–707 10.1002/ana.10042 11761467

[B73] Teplyuk N. M. Mollenhauer B. Gabriely G. Giese A. Kim E. Smolsky M. (2012). MicroRNAs in cerebrospinal fluid identify glioblastoma and metastatic brain cancers and reflect disease activity. Neuro Oncol. 14, 689–700 10.1093/neuonc/nos074 22492962 PMC3367845

[B74] Thomson D. W. Bracken C. P. Goodall G. J. (2011). Experimental strategies for microRNA target identification. Nucleic Acids Res. 39, 6845–6853 10.1093/nar/gkr330 21652644 PMC3167600

[B75] Trushina E. Dutta T. Persson X.-M. T. Mielke M. Petersen R. (2013). Identification of altered metabolic pathways in plasma and CSF in mild cognitive impairment and Alzheimer's disease using metabolomics. PLoS ONE 8:e63644 10.1371/journal.pone.0063644 23700429 PMC3658985

[B76] Tsuang M. T. Nossova N. Yager T. Tsuang M. M. Guo S. C. Shyu K. G. (2005). Assessing the validity of blood-based gene expression profiles for the classification of schizophrenia and bipolar disorder: a preliminary report. Am. J. Med. Genet. B Neuropsychiatr. Genet. 133B, 1–5 10.1002/ajmg.b.30161 15645418

[B77] Turchinovich A. Weiz L. Langheinz A. Burwinkel B. (2011). Characterization of extracellular circulating microRNA. Nucleic Acids Res. 39, 7223–7233 10.1093/nar/gkr254 21609964 PMC3167594

[B78] Ursini G. Bollati V. Fazio L. Porcelli A. Iacovelli L. Catalani A. (2011). Stress-related methylation of the catechol-O-methyltransferase Val 158 allele predicts human prefrontal cognition and activity. J. Neurosci. 31, 6692–6698 10.1523/JNEUROSCI.6631-10.2011 21543598 PMC6632869

[B79] Valadi H. Ekström K. Bossios A. Sjöstrand M. Lee J. Lötvall J. (2007). Exosome-mediated transfer of mRNAs and microRNAs is a novel mechanism of genetic exchange between cells. Nat. Cell Biol. 9, 654–659 10.1038/ncb1596 17486113

[B80] van Heerden J. H. Conesa A. Stein D. J. Montaner D. Russell V. Illing N. (2009). Parallel changes in gene expression in peripheral blood mononuclear cells and the brain after maternal separation in the mouse. BMC Res. Notes 2:195 10.1186/1756-0500-2-195 19781058 PMC2759952

[B81] Verderio C. Muzio L. Turola E. Bergami A. Novellino L. Ruffini F. (2012). Myeloid microvesicles are a marker and therapeutic target for neuroinflammation. Ann. Neurol. 72, 610–624 10.1002/ana.23627 23109155

[B82] Vickers K. Palmisano B. Shoucri B. Shamburek R. Remaley A. (2011). MicroRNAs are transported in plasma and delivered to recipient cells by high-density lipoproteins. Nat. Cell Biol. 13, 423–433 10.1038/ncb2210 21423178 PMC3074610

[B83] Villoslada P. Baranzini S. (2012). Data integration and systems biology approaches for biomarker discovery: challenges and opportunities for multiple sclerosis. J. Neuroimmunol. 248, 58–65 10.1016/j.jneuroim.2012.01.001 22281286

[B84] Wang K. Zhang S. Weber J. Baxter D. Galas D. (2010). Export of microRNAs and microRNA-protective protein by mammalian cells. Nucleic Acids Res. 38, 7248–7259 10.1093/nar/gkq601 20615901 PMC2978372

[B85] Wang Q. Li P. Li A. Jiang W. Wang H. Wang J. (2012). Plasma specific miRNAs as predictive biomarkers for diagnosis and prognosis of glioma. J. Exp. Clin. Cancer Res. 31, 97 10.1186/1756-9966-31-97 23174013 PMC3554474

[B86] Williams Z. Ben-Dov I. Elias R. Mihailovic A. Brown M. Rosenwaks Z. (2013). Comprehensive profiling of circulating microRNA via small RNA sequencing of cDNA libraries reveals biomarker potential and limitations. Proc. Natl. Acad. Sci. U.S.A. 110, 4255–4260 10.1073/pnas.1214046110 23440203 PMC3600502

[B87] Yang C. Wang C. Chen X. Chen S. Zhang Y. Zhi F. (2013). Identification of seven serum microRNAs from a genome-wide serum microRNA expression profile as potential noninvasive biomarkers for malignant astrocytomas. Int. J. Cancer 132, 116–127 10.1002/ijc.27657 22674182

[B88] Yao M. J. Chen G. Zhao P. P. Lu M. H. Jian J. Liu M. F. (2012). Transcriptome analysis of microRNAs in developing cerebral cortex of rat. BMC Genomics 13:232 10.1186/1471-2164-13-232 22691069 PMC3441217

[B89] Yuferov V. Nielsen D. A. Levran O. Randesi M. Hamon S. Ho A. (2011). Tissue-specific DNA methylation of the human prodynorphin gene in post-mortem brain tissues and PBMCs. Pharmacogenet. Genomics 21, 185–196 10.1097/FPC.0b013e32833eecbc 20808262 PMC3017726

[B90] Zhang R. Su B. (2009). Small but influential: the role of microRNAs on gene regulatory network and 3'UTR evolution. J. Genet. Genomics 36, 1–6 10.1016/S1673-8527(09)60001-1 19161940

[B91] Zhou L. Li X. Liu Q. Zhao F. Wu J. (2011). Small RNA transcriptome investigation based on next-generation sequencing technology. J. Genet. Genomics 38, 505–513 10.1016/j.jgg.2011.08.006 22133681

[B92] Zhubi A. Veldic M. Puri N. V. Kadriu B. Caruncho H. Loza I. (2009). An upregulation of DNA-methyltransferase 1 and 3a expressed in telencephalic GABAergic neurons of schizophrenia patients is also detected in peripheral blood lymphocytes. Schizophr. Res. 111, 115–122 10.1016/j.schres.2009.03.020 19386473 PMC3031301

[B93] Zovoilis A. Agbemenyah H. Agis-Balboa R. Stilling R. Edbauer D. Rao P. (2011). microRNA-34c is a novel target to treat dementias. EMBO J. 30, 4299–4308 10.1038/emboj.2011.327 21946562 PMC3199394

